# Using four decades of FDA orphan drug designations to describe trends in rare disease drug development: substantial growth seen in development of drugs for rare oncologic, neurologic, and pediatric-onset diseases

**DOI:** 10.1186/s13023-021-01901-6

**Published:** 2021-06-09

**Authors:** Kathleen L. Miller, Lewis J. Fermaglich, Janet Maynard

**Affiliations:** Office of Orphan Products Development, Office of the Commissioner, US Food and Drug Administration, 10903 New Hampshire Ave, Silver Spring, MD 20993 USA

**Keywords:** Oncology, Orphan drugs, Orphan Drug Act, Orphan drug designation, Pediatric, Rare disease, US Food and Drug Administration

## Abstract

**Background:**

Orphan drug designations are a useful proxy to investigate trends in rare disease drug development. Drug developers must receive a designation before they are eligible for the economic incentives of the Orphan Drug Act in the United States. We created a database of all orphan drugs designated between 1983 and 2019 that included numerous drug characteristics, including therapeutic area. In addition, we constructed a “broad disease” categorization of designations as an alternative to therapeutic area, based on disease etiology and age of onset rather than organ system. By looking at the pattern of orphan drug designations over the past four decades, this analysis studied the impact of the evolving rare disease drug development landscape and considers the future of rare disease therapies over the coming decades.

**Results:**

Between 1983 and 2019, a total of 5099 drugs and biologics received orphan drug designation. Designations more than doubled between the 1980s and 1990s, almost doubled between the 1990s and 2000s, and almost tripled in number between the 2000s and 2010s. The top three therapeutic areas represented in the orphan drug designations were: oncology (1910, 37%), neurology (674, 13%), and infectious diseases (436, 9%). The broad disease categorization found that the proportion of designations for pediatric-onset diseases has increased in the most recent decade to 27%.

**Conclusions:**

Analysis of the last four decades of orphan drug designation indicates seismic shifts have occurred in the rare disease drug development space. The number of designations granted more than quadrupled between the 1990s and 2010s. While these substantial increases led to growth in the absolute number of designations within all therapeutic areas (bar one) and broad disease categories, the relative proportions have seen considerable change over time. In the most recent decade, there have been notable increases in the proportion of drugs in oncology, pediatric-onset diseases, and neurologic disorders. The dramatic rise in overall orphan designations over the past four decades suggests we may continue to see an upward trajectory in designations leading to an increased number of approvals for drugs and biologics designed specifically for diagnosing, preventing, and treating rare diseases in the coming decades.

**Supplementary Information:**

The online version contains supplementary material available at 10.1186/s13023-021-01901-6.

## Introduction

In 1983, the United States Congress passed the Orphan Drug Act (ODA) to incentivize the development of drugs for rare diseases, defined in the ODA as affecting fewer than 200,000 people in the US. These incentives were primarily financial, to stimulate the biopharmaceutical industry’s interest in developing drugs for the relatively small populations of patients affected by these diseases, many of which are debilitating or life-threatening [1]. These incentives currently include a 25% tax credit on applicable research and development expenditures, waived user fees when submitting applications to the Food and Drug Administration (FDA), and the potential for a seven-year period of orphan drug exclusivity for the approved rare indication [2].

While it is clear from previous research that the incentives created by the ODA have played a major role in inducing investment in rare disease drug development, there are other factors at work as well that have stimulated development in this field.[3–7]

At the time the ODA was passed, most drugs being approved were small molecules developed for diseases with large patient populations, such as gastroesophageal reflux disease, hypertension, and bacterial infections [8]. However, since the passage of the ODA, we have seen dramatic advances in basic and translational science [9]. These scientific advances over the past four decades, including the sequencing of the human genome and a better understanding of disease processes at a molecular level, have led to novel therapies that can significantly alter the disease state of patients [10–12].

Many of the most groundbreaking therapies that have emerged in the last decade have been those developed to treat rare diseases, partly because the characteristics of rare diseases lend themselves to being a starting point in the translation of these new scientific discoveries to clinical medicine [13, 14]. In addition to an overall increased interest in rare disease product development, these scientific advances, along with other factors, may impact the therapeutic areas and types of products being developed for rare diseases.

However, advances in science are not the only factor influencing this interest in rare diseases. There have been significant changes in the way drugs are priced and reimbursed since the passage of the ODA [15]. We have also seen the creation of active patient groups for rare diseases, many of whom play a central role in creating patient registries and centers of excellence, and who fund the development of diagnostics, devices and drugs for their conditions [1, 16–18].

Despite these advances, and a marked increase in rare disease product approvals, the vast majority of the estimated 7000 known rare diseases still do not have approved therapies [4]. To optimally support the development of products for rare diseases, it is critical to understand the evolving trends in this area. We analyzed how rare disease drug development has changed over the last four decades by quantitatively studying the drugs that have received orphan drug designation since the ODA was enacted.

The orphan drug designation is administered by the Office of Orphan Products Development (OOPD) within the FDA. Drug developers must request and be granted designation before they are eligible for any of the ODA designation incentives. (The ODA also created the orphan product clinical trial grant program, but drugs do not need to be designated to apply for this incentive.) Drug sponsors submit a request for designation to the OOPD by presenting evidence that they are developing the drug for a rare disease and demonstrating the scientific rationale of the drug (clinical or preclinical evidence that establishes the medically plausible basis for the use of the drug in that rare disease) [19].

Orphan drug designation is a useful proxy for total rare disease drug development because it likely captures the majority of commercial development that is occurring and can be applied for at any time before the submission of a marketing application. By looking at the pattern of designations over the past four decades, we are able to analyze the impact of the changing rare disease drug development landscape and consider the future of therapeutic, preventive, and diagnostic agents for rare diseases over the coming decades.

## Methods

We gathered all orphan drug designations from 1983 to 2019 from an internal FDA database. Designation information is also publicly available via the searchable list on the FDA webpage (The final dataset is provided in the Additional file 1: Data Appendix) [20]. These data capture multiple characteristics of the designation, including: date of designation, disease or condition that the drug was designated for, and whether there have been any approvals associated with the designation. An orphan drug may have multiple approvals associated with it, including for new indications or formulations [4].)In this analysis, we often present “approvals” as designations associated with at least one approval to more accurately elucidate the unique number of designations with approvals, without the confounding effect of multiple approvals associated with one designation.

Using an associated internal FDA database, we captured whether the product was a small molecule drug or a biologic product, and whether it was being used as a treatment, preventive, or diagnostic agent for the designated disease.

To add to these characteristics, the designations were classified into two additional categories based on the rare disease being treated. First, we grouped designations into a traditional therapeutic area category. Classification was based on the organ system that was primarily affected by the underlying disease (e.g., sickle cell disease is primarily a blood disorder so was categorized as hematologic). Therapeutic area categorization also included more general categories (e.g., oncology, infectious disease) which were prioritized over the system-based classification (e.g., gastric cancer was categorized as oncology rather than gastrointestinal).

Second, we constructed an alternative categorization of designations based on a definition of disease using disease etiology and typical median age of onset greater than (adult-onset) or less than (pediatric-onset) 18 years old. Designations were classified into one of six “broad disease” categories: oncologic diseases (“oncology”), infectious diseases, pediatric-onset diseases (“pediatric-onset”), adult-onset diseases (“adult-onset”), emergency-related conditions (“emergency”), or transplant-related conditions (“transplant”).

Our construction of a broad disease categorization contributes to the assessment of FDA orphan drug designations through multiple dimensions. A first contribution is the addition of the age of onset of a disease into the categorization, which allows us to analyze rare disease drug development trends through this lens. Separating adult-onset, pediatric-onset, and age-agnostic diseases allows us to determine where development trends are occurring and whether there is limited development in any of these areas, an analysis that is not possible when viewing the data within the traditional categorization by therapeutic area.

Second, the broad disease categorization allows us to view diseases outside of the single system framework of the traditional therapeutic area. Many rare diseases affect multiple systems or multiple organs, and therefore categorizing them into one exclusive therapeutic area does not account for the full range of affected organ systems. For example, cystic fibrosis is often categorized into the “pulmonary” therapeutic area, yet it affects multiple organ systems, and is more holistically classified as an inherited or early-onset genetic disease. Additionally, comparisons between therapeutic areas can suffer from mixed foundational comparative bases (e.g., comparing oncologic disease, a disease process, with ophthalmologic disease, a disease location). By focusing on etiology rather than organ system, the broad disease categorization allows for an alternative view of the designations that is commensurate and encompassing.

To construct the broad disease categorization, we utilized the following methodology. Similar to the therapeutic area categorization hierarchy, the broad disease category oncology was prioritized over the other five categories to account for its unique size in rare disease drug development. The infectious disease, emergency, and transplant categories were prioritized over pediatric- and adult-onset to account for their age-agnostic characteristics. These three non-oncology age-agnostic categories were created using a qualitative analysis of the data to determine the appropriate broad disease categories for these data. Examples of such diseases and conditions included: soil-transmitted helminthiasis (infectious disease), snake bite (emergency), severe burns (emergency), and graft-vs-host disease (transplant).

Additionally, designations for conditions that were complications of underlying diseases, side effects of treatments for the underlying diseases, or opportunistic diseases primarily associated with underlying diseases (e.g., HIV/AIDS) were categorized according to the broad disease category for the underlying disease.

## Results

There were 5099 orphan drug applications designated between 1983 and 2019. As of December 31, 2019, 724 (14%) of these designations had at least one associated approval, and there were 878 total approvals. For first approvals, 35% occurred within two years of designation, 69% occurred within five years, and 92% occurred within ten years.

Of these orphan products, 59% (3010) were small molecules and 41% (2089) were biologics. This proportion stayed relatively constant when evaluated by decade. Treatments accounted for 92% (4678) of the designations, preventives 7% (332), and diagnostics 2% (89).

**Fig. 1:**
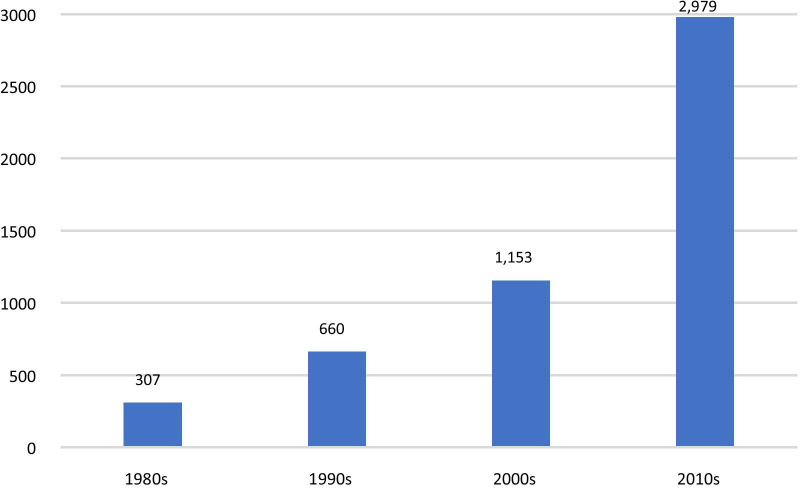
Count of FDA orphan drug designations by decade, 1983—2019. 1980s includes 1983–1989

Designations more than doubled between the 1980s and 1990s, almost doubled between the 1990s and 2000s, and almost tripled in number between the 2000s and 2010s (Fig. 1).

**Fig. 2:**
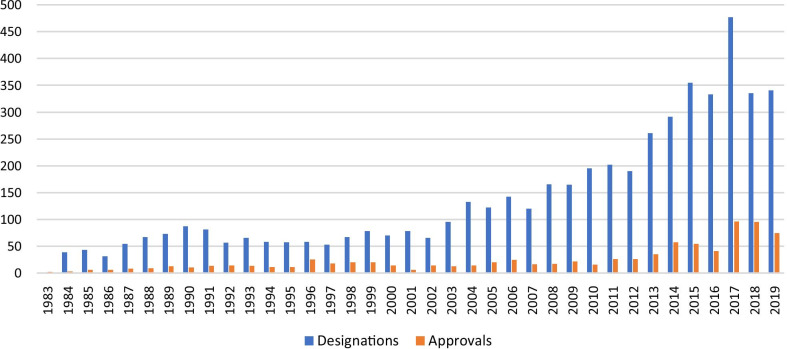
Count of FDA orphan drug designations and total approvals by year, 1983–2019

Between 1983 and 2012, there appeared to be a cyclical trend in number of designations, with multiple peaks and troughs and a general increase in the magnitude of the cycles since 2002 (Fig. 2). However, since 2013, we have seen a strong upward trend in the number of designations. There were more designations in 2017 than any other year, the cause of which is described in the discussion.

### Therapeutic area categories

**Fig. 3:**
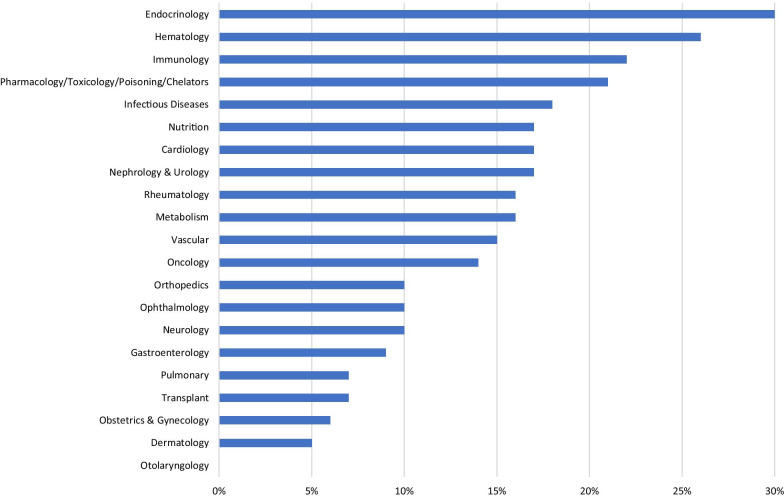
Distribution of FDA orphan drug designations with at least one approval within therapeutic area, 1983–2019

The top five therapeutic areas represented in the orphan drug designations were: oncology (1910, 37%), neurology (674, 13%), infectious diseases (436, 9%), metabolic disorders (280, 5%), and hematology (254, 5%) (Table 1). In contrast, the top five therapeutic areas with designations with at least one associated approval that had the highest percentage of approvals per designations were: endocrinology (30%), hematology (26%), immunology (22%), pharmacology & toxicology (21%), and infectious disease (18%). (Fig. 3).

**Table 1:** FDA orphan drug designations and approvals by therapeutic area, 1983–2019

Therapeutic area	# of designations	% of total designations	# with at least one approval	% of designations with at least one approval
Oncology	1910	37	267	14
Neurology	674	13	69	10
Infectious diseases	436	9	79	18
Metabolism	280	5	45	16
Hematology	254	5	65	26
Transplant	207	4	14	7
Pulmonary	206	4	15	7
Gastroenterology	195	4	17	9
Ophthalmology	153	3	16	10
Endocrinology	140	3	42	30
Vascular	135	3	20	15
Rheumatology	119	2	19	16
Dermatology	94	2	5	5
Pharmacology and toxicology & poisoning and chelators	87	2	18	21
Immunology	54	1	12	22
Nephrology and urology	54	1	9	17
Cardiology	41	1	7	17
Orthopedics	31	1	3	10
Obstetrics and gynecology	17	< 1	1	6
Otolaryngology	6	< 1	0	0
Nutrition	6	< 1	1	17

**Fig. 4:**
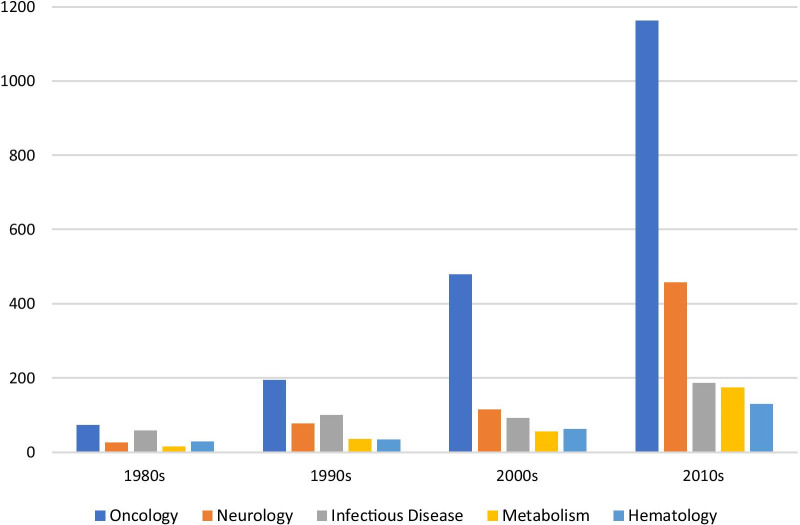
Count of FDA orphan drug designations within therapeutic area by decade, 1983—2019. Only top five therapeutic areas with greater than 250 total designations were included in Fig. 4. Please see the Additional file 2: Table for a full presentation of the trends within therapeutic area over time

These proportions have changed over the decades (see Additional file 2: Table for a full presentation of these results). In the 1980s, oncology represented 24% (73) of designations, but in the 2010s that proportion had grown to 39% (1163). (Fig. 4) There was an even greater proportional increase in neurology, which almost doubled from 8% (26) of designations in the 1980s to 15% (457) in the 2010s. This near doubling was the second highest proportional increase of the therapeutic areas; rheumatology was the highest going from < 1% (1) in the 1980s to 3% (79) in the 2010s. Conversely one of the steepest proportion decreases was in infectious diseases, which in the 1980s represented 19% (58) of designations, but in the 2010s had decreased to 6% (186) (although the absolute number of designations in this therapeutic area increased over this period).

### Broad disease categories

Our categorization by broad disease found that oncology represented 37% (1896) of the total designations, pediatric-onset 25% (1278), adult-onset 23% (1166), infectious diseases 8% (418), transplant 4% (211), and emergency 3% (130). (Note that these absolute numbers for oncology and infectious diseases differ slightly from the therapeutic area categorization due to the different criteria by which these designations were classified.)

**Fig. 5:**
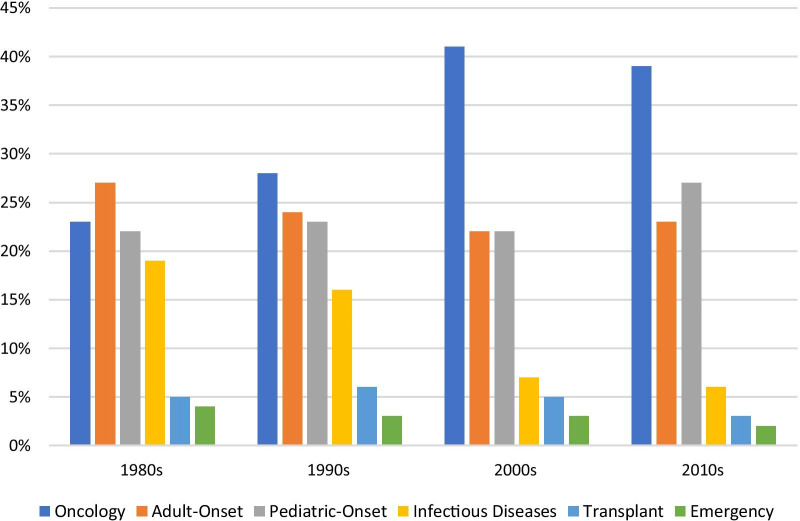
Distribution of FDA orphan drug designations within broad disease category by decade, 1983—2019

The proportion of designations representing four of these categories has changed markedly over the decades (Fig. 5). (Except for the emergency and transplant categories, which have remained at or below 5% of designations through the whole period.) The first major change is that the proportion of infectious disease designations declined precipitously over the study period (although the absolute number of these designations actually rose, as can be seen in Fig. 4).

For the last three categories, we have seen varying fluctuations over the last four decades. The proportion of pediatric-onset, adult-onset, and oncology designations were relatively the same in the 1980s. In the 1990s, the proportion of pediatric-onset stayed relatively stable, while adult-onset somewhat decreased and oncology somewhat increased. In the 2000s, the proportion of both pediatric-onset and adult-onset diseases had decreased to almost 20% each, while oncology designations had increased to over 40%. In the 2010s, pediatric-onset designations spiked to 27% while adult-onset designations remained relatively stable.

**Fig. 6:**
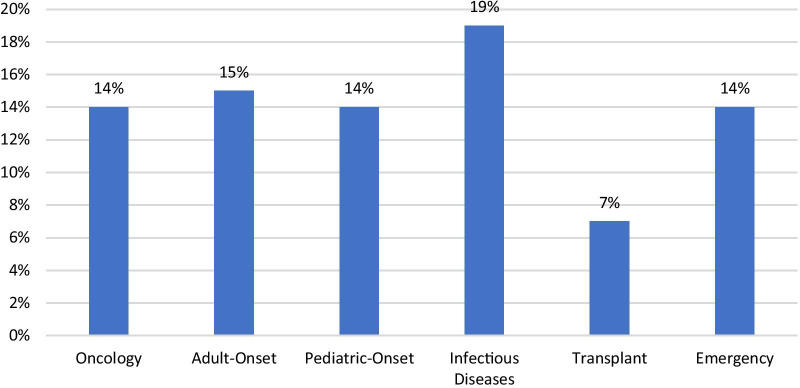
Distribution of FDA orphan drug designations with at least one approval within broad disease category, 1983—2019

We also investigated the number of designations with at least one approval within each broad category (Fig. 6). The proportion of designations with at least one approval was very similar for four of the categories, at 14–15%. Transplant drugs were much lower at 7%, while infectious diseases were a third higher at 19%.

## Discussion

Since the ODA was enacted in 1983, over 5000 drugs and biologics have received orphan drug designation and a new era of rare disease biopharmaceutical innovation and investment has been reached. In just ten years since the last quantitative analysis performed by OOPD, designations and approvals for rare disease drugs have nearly tripled [21]. These recent increases in rare disease drug development have also been observed in the European Union (EU) and Japanese orphan drug programs, indicating that these are international trends [22–24].

This increase in designations also appears to have translated into an increase in approvals, with a similar trend appearing to occur in the EU [4, 24]. However, it is important to note that this may occur on a substantial lag due to the timeline of development: orphan designations may be granted at any stage of the development process, from the preclinical phase up to before a marketing application is submitted [25, 26]. This limits the interpretability of the time from designation to approvals, as well as when designations may translate into approvals in the future. Regardless, the next decade may see a large increase in rare disease drug approvals, if this complementary relationship between designations and approvals persists. Projecting when these future approvals may occur, and in what disease areas we may see them, is an important area of future study [27].

The notable spike in designations in 2017 was due to the Orphan Drug Modernization Plan, which streamlined the review process, reviewed all pending designation requests, and created a goal of a 90-day review timeline for requests [28]. The outcome of this effort was an increase in designations granted for 2017, due to the processing of all orphan drug designation requests that were older than 120 days, and the more rapid review of incoming applications.

A second pronounced change was the large increase in the number of designations granted for oncology drugs. In the 1980s, there were 73 oncology designations, and by the 2010s, that number had risen to 1163, almost a 1500% increase. Their share of total designations has risen as well, from 24 to 39%. These increases seem likely to be primarily driven by advances in cancer science, which have translated into numerous drug development programs for rare cancers [5]. This is not surprising, as funding for basic science research in oncology in the US, primarily through the National Cancer Institute at the National Institutes of Health, has been supported for decades, including a push for a “Cancer Moonshot” [29]. OOPD also sponsors grants for rare disease clinical trials research, including for rare cancers [30].

Within the therapeutic areas, we also found two interesting trends. First, there was a sizable decrease in the proportion of designations of drugs developed for infectious diseases (from 19% in the 1980s, the second highest therapeutic area category behind oncology, to 6% in the 2010s, tied for the third highest) (Additional file 2: Table) However, the absolute number of designations did not decrease, in fact, there was an over 200% rise (from 58 to 186). While current infectious disease-specific incentives, such as the tropical disease priority review voucher and the Generating Antibiotic Incentives Now (GAIN) Act, may be stimulating some of this increase, they do not seem to be enough to make up for the tremendous growth in rare disease drug development in other therapeutic areas.[31–33] However, while the proportional decrease is striking, the large increase in the absolute number of designations implies that there should not be substantial concern regarding development and innovation in this space.

Second, we saw an almost doubling in the proportion of neurology orphan drug designations between the 1980s and 2010s, from 8 to 15% of all designations. This was correlated with a tremendous increase (26 to 457) in the absolute number of designations. While it is not clear precisely what factors in the rare disease drug development landscape may be the cause of the increase, given the amount of unmet need in this space, it is clear that any increase in development is welcomed by patients and other stakeholders [34].

The results of the broad disease category analysis indicate the wide breadth of diseases considered rare under the ODA definition. Many might be surprised that the age-agnostic diseases include categories for transplant conditions (e.g., graft-versus-host-disease, transplant surgery complications) and emergency conditions (e.g., burns, poisonous animal envenomation). While these two categories have relatively few designations associated with them, both are distinct and important.

It may also be surprising that pediatric-onset diseases represent only a quarter of all designations. Many of the most well-known rare diseases are pediatric-onset, such as cystic fibrosis, Duchenne muscular dystrophy, and sickle cell disease [35]. The development of drugs for pediatric-onset diseases appears to be rising: in the 2010s they represented 27% (811) of all designations, the highest proportion and absolute number for the pediatric-onset broad disease category, from any decade. Therefore, while pediatric-onset diseases are not the only group of rare diseases being studied by industry, there is substantial and apparently increasing relative investment being made in this space.

Interestingly, when analyzing the results of designations with at least one approval, we find a much larger variation within therapeutic area (0%-30%) than we do within broad disease category (7%-19%). This variation may be due to more validated and predictive efficacy biomarkers within certain organ systems, driving the difference within therapeutic area categories.[36–38] It may also indicate the wide variety of diseases studied within pediatric-onset and adult-onset broad disease categories, apparently leading to an averaging out of approval proportions.

Lastly, we note the interesting finding that the proportion of designated biologics has remained constant over the decades. This is surprising given that, over the same period, the proportion of biologic orphan products being approved has increased [4]. This indicates that orphan biologics may have a higher rate of approval success when compared to small molecule products. It is possible that this is due to specific properties of some biologics, such as specificity for the pathogenesis of a specific rare disease, or factors such as the types of sponsors developing biologics and their experience within the rare disease space [39]. However, it is important to note that the composition of the types of moieties in these two groups has changed over the past four decades, which could also have contributed to these differences. For example, in recent years, there have been increased designations for cannabinoids (small molecules) and gene therapies such as antisense oligonucleotides (biologics). Further studies would be needed to fully elucidate the causes of this difference.

Future research on FDA orphan drug designations may also wish to: investigate the types and degree of innovation of the drugs and biologics being designated, determine the specific diseases being designated, and conduct a detailed analysis of the orphan-designated approvals.

### Limitations

There are several limitations to this study. First, orphan designations are not a perfect proxy for the full drug development landscape for rare disease. Some companies, nonprofits, or academic centers conducting research on rare disease drugs may not apply for orphan designation. This may also be true for drugs being developed outside the US. Additionally, while many orphan products are designated in the preclinical phase, some are not designated until the early-to-mid clinical phase. However, we believe that the products that receive orphan designation represent a large enough proportion of the rare disease drug development landscape, and therefore, analysis and projection to the larger market are appropriate.

We also acknowledge that the proportion of orphan products with at least one approval suffers from a lag bias. Drugs designated in the most recent ten years (which is a large proportion of the total) may still be in development, and it would be too early to expect them to have been approved [40]. However, we still believe that this measure is useful and of interest.

Lastly, our categorization of therapeutic area and broad disease includes a degree of subjectivity. We attempted to ameliorate this by having the primary determinations reviewed by a second author and discussing discrepancies. However, some level of subjectivity is still inherent in the categorization. Additionally, because the broad disease categorization is a construction unique to this paper, it has limited comparability to previous studies on orphan drug designations. The use of therapeutic area as the primary categorization ameliorates this limitation and the results of this portion of the analysis can be compared to previous work [21, 25, 41, 42].

## Conclusion

Analysis of the last four decades of orphan drug designation indicate seismic shifts have occurred in the rare disease drug development space. The number of designations granted have more than quadrupled between the 1990s and 2010s, indicating explosive growth in the industry interest in rare disease drugs since the ODA was enacted. While all six broad disease categories have increased in absolute terms, the proportion of rare oncologic drugs being developed has increased substantially, as has the proportion of drugs for pediatric-onset diseases. We also see proportionally large increases in the neurology therapeutic area. The dramatic rise in overall orphan drug designations over the past four decades suggests we may continue to see an upward trajectory in designations leading to an increased number of approvals for drugs and biologics designed specifically for diagnosing, preventing, and treating rare diseases in the coming decades.

## Supplementary Information

**Additional file 1.** Data Appendix.

**Additional file 2.** Table.

## Data Availability

The datasets generated and analyzed during the current study are publicly available as an appendix to this manuscript.

## Abbreviations

FDA: US Food and Drug Administration
ODA: Orphan Drug Act
OOPD: Office of Orphan Products Development

## Declarations

### Disclaimer

This article reflects the views of the authors and should not be construed to represent FDA’s views or policies.
