# Supplementary material for: Using four decades of FDA orphan drug designations to describe trends in rare disease drug development: substantial growth seen in development of drugs for rare oncologic, neurologic, and pediatric-onset diseases
Source: Orphanet J Rare Dis. 2021 Jun 9;16:265. doi: 10.1186/s13023-021-01901-6 (PMC8191002; doi:10.1186/s13023-021-01901-6)
Supplement: Supplementary file 2 — Additional file 2. Table. [file 13023_2021_1901_MOESM2_ESM.docx]

*Supplemental Table. Proportion of Designations by Therapeutic Area by Decade*

| **Therapeutic Area** | **1980s** | **1990s** | **2000s** | **2010s** |
| --- | --- | --- | --- | --- |
| Cardiology | 1% | 1% | 1% | 1% |
| Dermatology | 2% | 2% | 2% | 2% |
| Endocrinology | 8% | 3% | 2% | 2% |
| Gastroenterology | 3% | 3% | 4% | 4% |
| Hematology | 9% | 5% | 5% | 4% |
| Immunology | 1% | 2% | 1% | 1% |
| Infectious Diseases | 19% | 15% | 8% | 6% |
| Metabolism | 5% | 5% | 5% | 6% |
| Nephrology & Urology | 2% | 2% | 1% | 1% |
| Neurology | 8% | 12% | 10% | 15% |
| Nutrition | 1% | <1% | <1% | 0% |
| Obstetrics & Gynecology | <1% | <1% | <1% | <1% |
| Oncology | 24% | 30% | 42% | 39% |
| Ophthalmology | 4% | 2% | 2% | 3% |
| Orthopedics | <1% | 1% | 1% | 1% |
| Otolaryngology | 0% | <1% | <1% | <1% |
| Pharmacology & Toxicology & Poisoning & Chelators | 4% | 2% | 2% | 1% |
| Pulmonary | 4% | 6% | 5% | 3% |
| Rheumatology | <1% | 2% | 2% | 3% |
| Transplant | 5% | 5% | 5% | 3% |
| Vascular | 1% | 1% | 3% | 3% |
